# Medical Certificates in the Daily Papers

**Published:** 1878-01

**Authors:** 


### Medical Certificates in the Daily Papers.

It has often been said that it is a reflection upon the medical profession to have a Code of Ethics; that the very existence of such a set of laws implies a temptation for wrong-doing, and a necessity for discipline. Every medical man is forced to admit the truth of this, but he has some comfort in the thought that to err is human.

The medical, as far as the Code is concerned, is different from any other equally liberal profession. In no other calling is there any system of laws by which the actions of the individual members are governed. This does not, however, prove that there is less necessity for standard rules of conduct and of government either in the clerical or legal profession, but that the profession of medicine is in advance of the others, and is more zealous in preserving its dignity, in developing its own resources, in protecting the rights of its individual members, and enlarging its sphere of usefulness. The proposition so often made, to dispense with the Code, is certainly far from a reasonable one. The more we see of medical men in their relations to each other and to the profession, the more we are convinced of the necessity of some definite rules to govern their conduct. This is especially so of late years, and the conclusion seems almost irresistible that the moral tone of the profession has very much lowered in that time. To what particular cause this may be due, we are at a loss to determine. We wish barely to allude, in this connection, to the fact that very many of our leading men are boldly doing what they would not have thought of doing years ago. The illustration in point which will occur to every reader is the very objectionable and shameful parade in the daily papers of medical certificates in the advertisements of certain mineral waters. If their example is to be followed, and a new fashion instituted, the dignity and influence of medicine will be at an end, and the public will have no means of distinguishing those whom we would gladly honor, from the unprincipled quacks, whose only concern is to be kept in public view.

Advertising of any and every sort is very properly condemned by the profession. The reason for this is obvious, in that it allows a few to take advantage of the many. There is no intention of preventing physicians from holding intercourse with the public as teachers. In that capacity they can speak for their profession, and help to maintain its dignity,honor and usefulness; but as soon as they place themselves before the public as practitioners, that moment they lay themselves open to the charge of advertising. It is certainly not educating the public, to certify to the fact Appolinaris water is "a delightful beverage," or is "far superior to Vichy,Seltzer, or any other;" or "is most grateful and refreshing;" or "is useful and agreeable;" or "healthful and well suited for dyspepsia;" or "by far the most agreeable alone or mixed with wine. and useful in catarrhs of the stomach or bladder, or in gout;" or "well suited for dyspepsia and cases of acute disease;" or lastly, "is not only a luxury, but a necessity." And yet these very remarkable certificates, with the names and addresses of the writers, appear in the papers all over the country without any open protest being made against it. If these gentlemen are thus doing their duty or elevating their profession in the eyes of the public—educating the people to a proper appreciation of the dignity of a noble calling and the value of medical certificates—it is time that a new code should be made. As we have previously remarked, the profession is much interested concerning the abstract justice of this method of advertising. There is not so much concern in regard to the individuals, as to the principle of which they are seemingly the willing exponents. If the transgression was made by ordinary medical men, charges would have been preferred; they would be branded as advertisers, their names be omitted from the *Medical Register*, and if non-repentant after a reprimand, they would be expelled from their County Society.

Again, in the advertisement of another mineral water—the Hunyadi Jànos—medical certificates from the same gentlemen appear. In reading them, one can hardly escape the conviction that no other laxative should be used; so agreeable, painless and certain is it; in fact, that it is the only one specially "adapted for daily use." Who, now, can blame any reader of a daily paper if he quotes the highest medical authority to prove that this mineral water is better than anything else? Certainly, if these certificates were written to order, they could not help the sale of these proprietary waters more effectually. But the question is not how much of the waters may or may not be sold, but how far it is proper for medical men to publicly recommend their use, as in dyspepsia, catarrhs of the bladder and stomach, in gout, etc. ? At best, this is cheapening medical advice by offering it gratis, by placing a premium on self-medication, by encouraging indiscriminate prescribing, and by lowering the tone and dignity of the profession generally. If these certificates were for circulation among medical practitioners, they would be taken at their real value; but paraded before the public, they are apt to mislead, and create misapprehension as to the legitimate resources of our materia medica. If there is any excuse for an error in judgment in giving the certificates, there is certainly none for allowing the daily advertisement of their names as medical practitioners in the different daily papers. It is somewhat astonishing, knowing, as they must, that their course was not indorsed by their brethren, these gentlemen should not long ago have withdrawn permission to use their names in every daily paper, and thus have vindicated the true dignity and higher instincts of a time-honored profession. •But as their case now stands, they have not only failed to do so for many months, but the continual appearance of their names in the public advertisements would seem to defy that discipline which with lesser men would have been swift and certain.

We repeat that the profession is very much interested in this question, and would like to see it settled one way or the other. We have on a previous occasion brought it to the notice of the County Society, and now that its new Committee of Ethics has been appointed, may we not hope that some authoritative decision will be rendered ?—*Medical Record.*

